# Supplementary material for: A benchmarked comparison of software packages for time-lapse image processing of monolayer bacterial population dynamics
Source: Microbiol Spectr. 2024 Jul 9;12(8):e00032-24. doi: 10.1128/spectrum.00032-24 (PMC11302142; doi:10.1128/spectrum.00032-24)
Supplement: Supplemental material — Supplemental table and figures. [file spectrum.00032-24-s0001.pdf]

Supplemental material for ‘A benchmarked comparison of software packages for time-lapse image processing of monolayer’ bacterial population dynamics, Ahmadi et al.

Supporting Information Text

1. Image processing package overviews

Table S1. Features of the software packages evaluated in this study

Feature	CellProfiler	SuperSegger	DeLTA	FAST
Pre-processed inputs	✓	×	×	✓
GUI	✓	✓	×	✓
Online segmentation correction	✓	×	×	×
Online tracking correction	×	×	×	✓
Cell type	eukaryote/prokaryote	prokaryote	eukaryote/prokaryote	prokaryote
Language	python	MATLAB/C++	python	MATLAB
Release date	2006	2016	2020	2021
Online discussion forum	✓	×	✓	×

2. Results

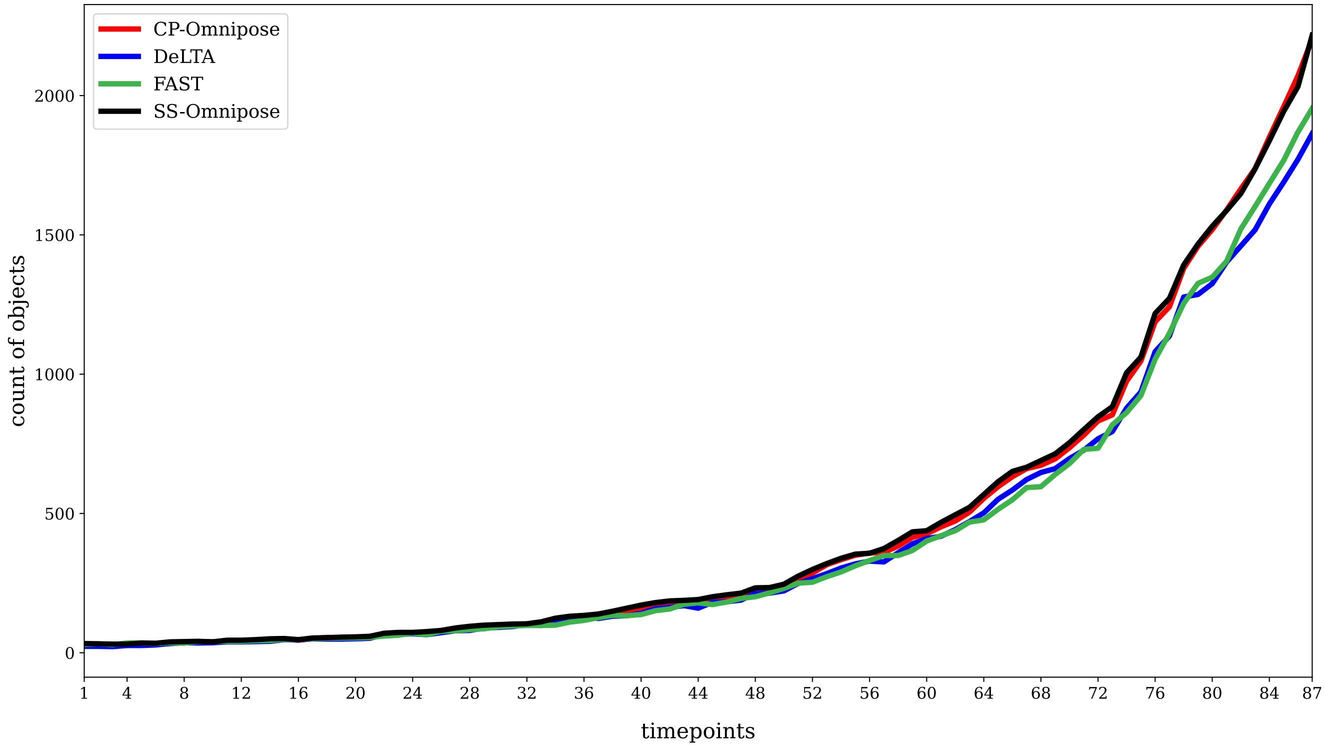

Fig. S1. Segmentation object count results for time-lapse of unconstrained *X. campestris* growth. Ground truth was not generated for this dataset. The time interval between each frame is 3 minutes, and the total duration of the experiment is 258 minutes. Full dataset at Github (CP: CellProfiler, SS: SuperSegger)

A. Segmentation object counts for *X. campestris*.

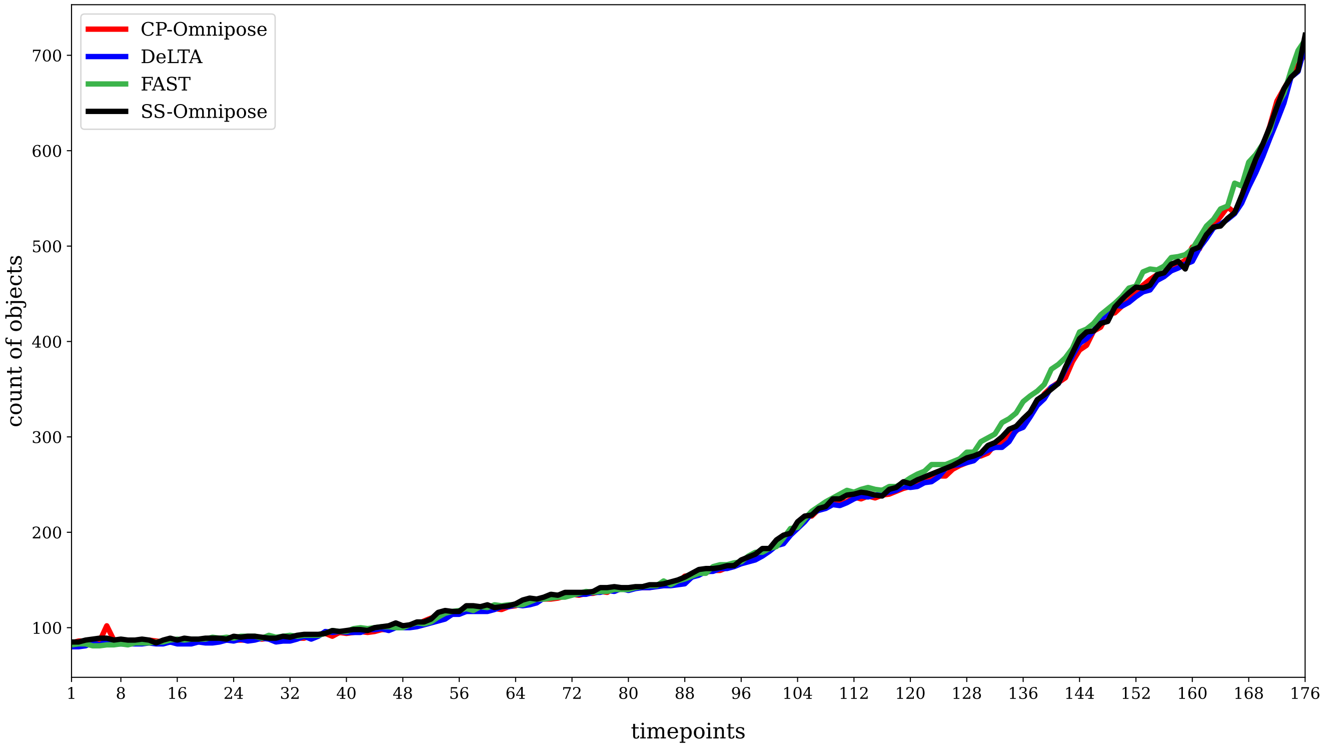

Fig. S2. Segmentation object count results for time-lapse of unconstrained *P. putida* growth. Ground truth was not generated for this dataset. The time interval between each frame is 3 minutes, and the total duration of the experiment is 528 minutes. Full dataset at Github. (CP: CellProfiler, SS: SuperSegger)

B. Segmentation object counts for *P.putida*.

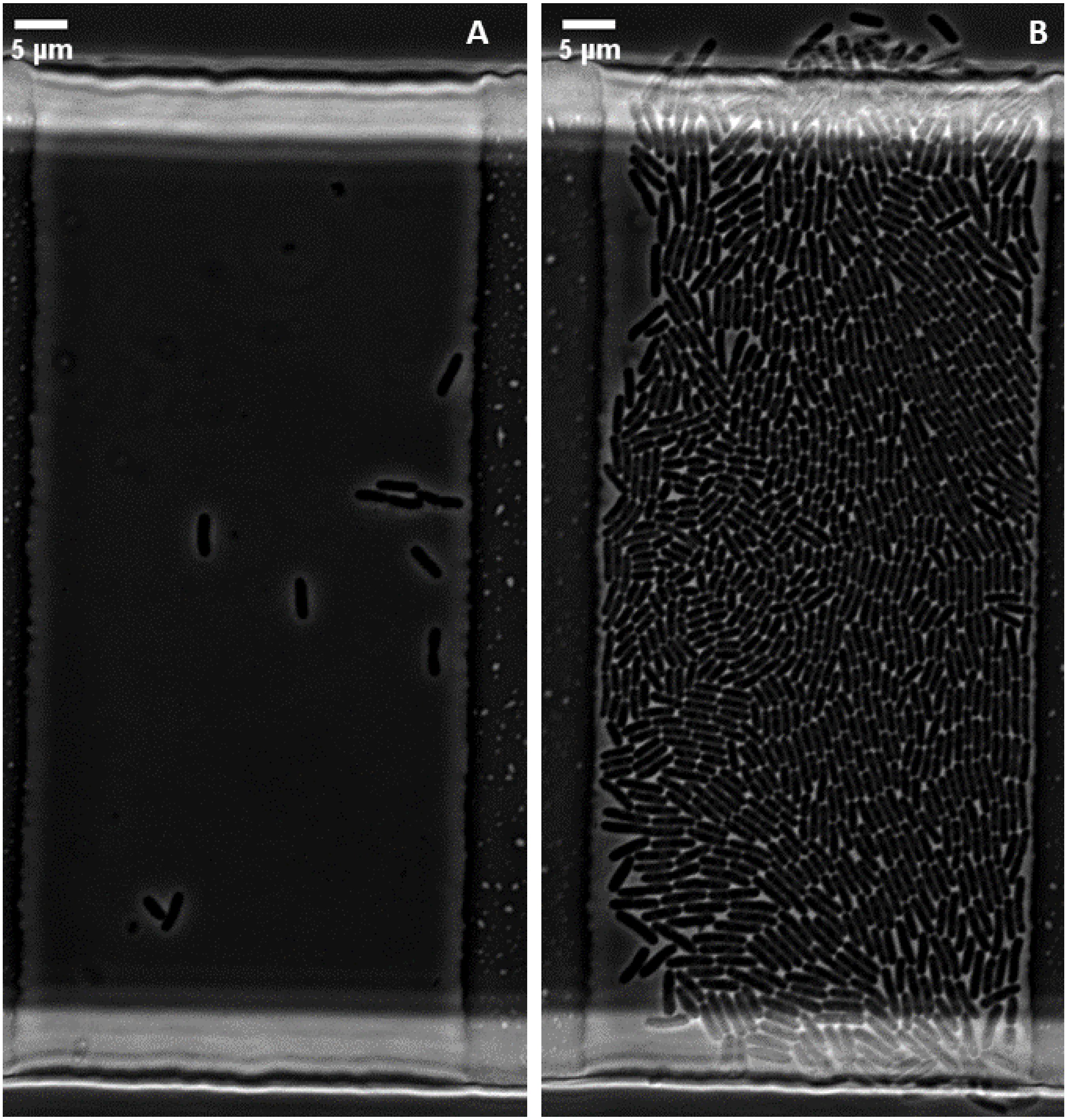

Fig. S3. *E.* coli growth in a constrained environment. (A) First and (B) last (54th) timestep of *E.coli* growth in a microfluidic trap. The initial frame contains 10 cells. The population grows to 1049 cells by the 54th timestep, after 162 minutes. A distinct halo of increased brightness can be observed near the chamber entrances; this can be attributed to imaging artifacts arising from the neighbouring (taller) flow channel.

C. First and last timepoints of microfluidic time-lapse.

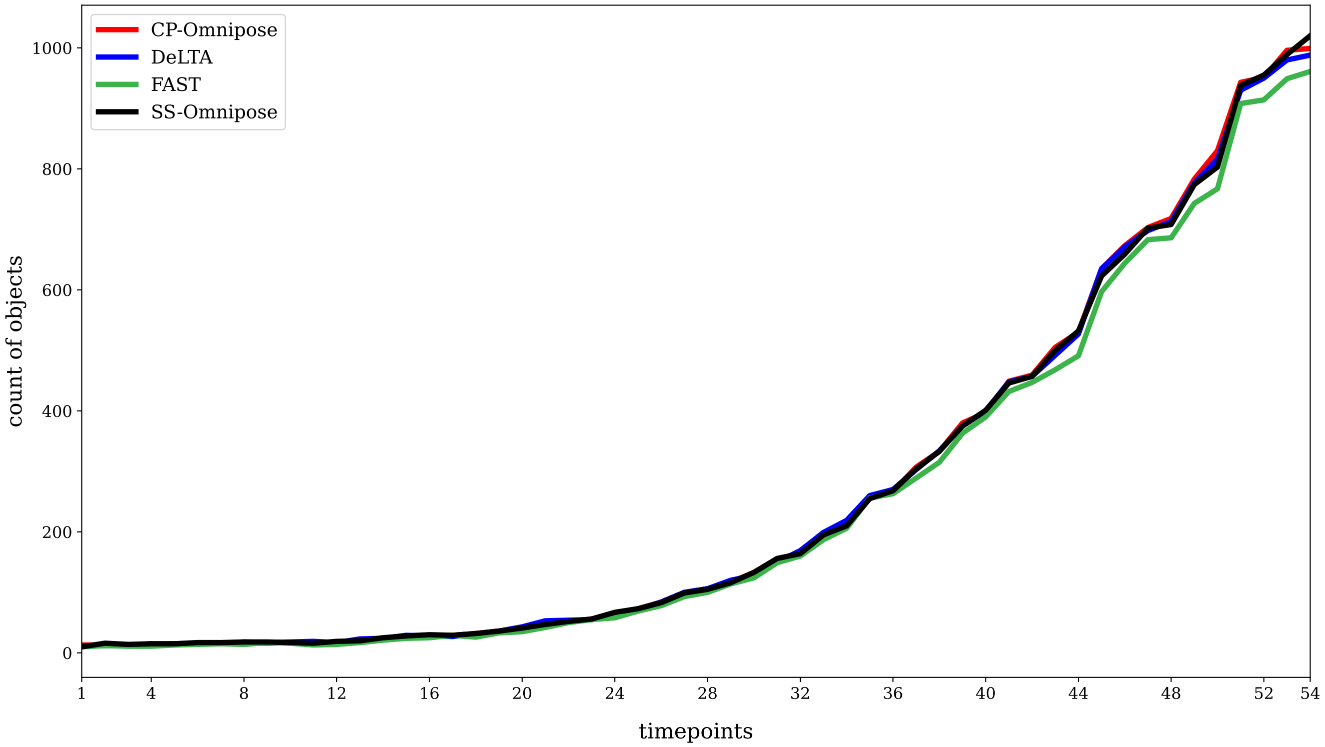

Fig. S4. Segmentation object count results for time-lapse of constrained *E.coli* growth. Ground truth was not generated for this dataset. The time interval between each frame is 1.5 minutes, and the total duration of the experiment is 81 minutes. (CP: CellProfiler, SS: SuperSegger)

D. Segmentation object counts for *E.coli* in the microfluidic trap.

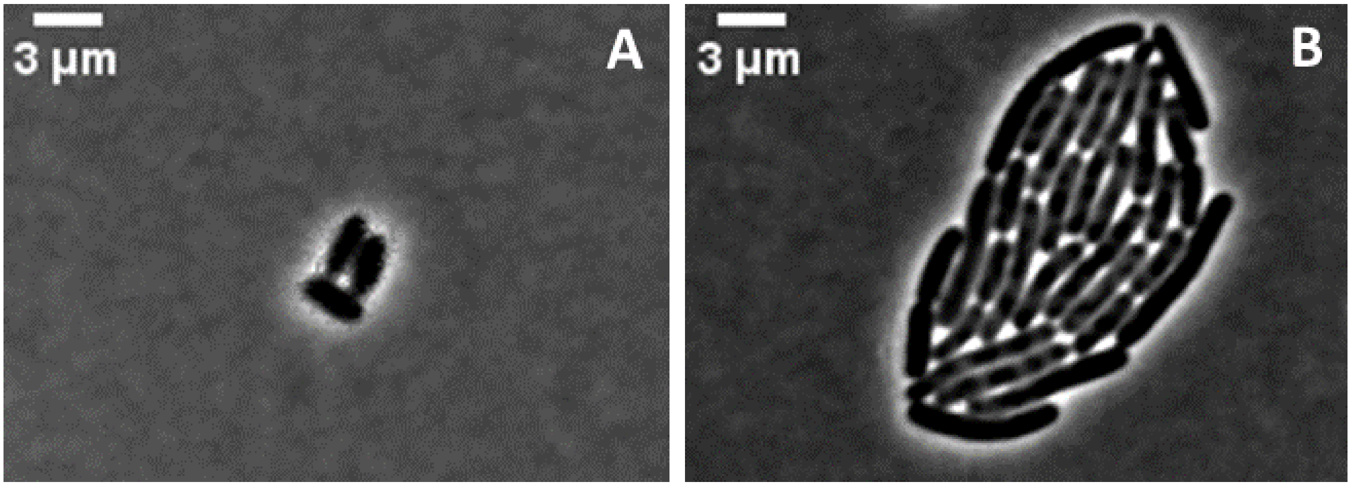

Fig. S5. (A) First and (B) last (31st) frame of a cropped time-lapse of unconstrained *E.coli* growth. The time interval between each frame is 5 minutes, and the total duration of the experiment is 150 minutes.

E. Representative images from the workable *E.coli* time-lapses.

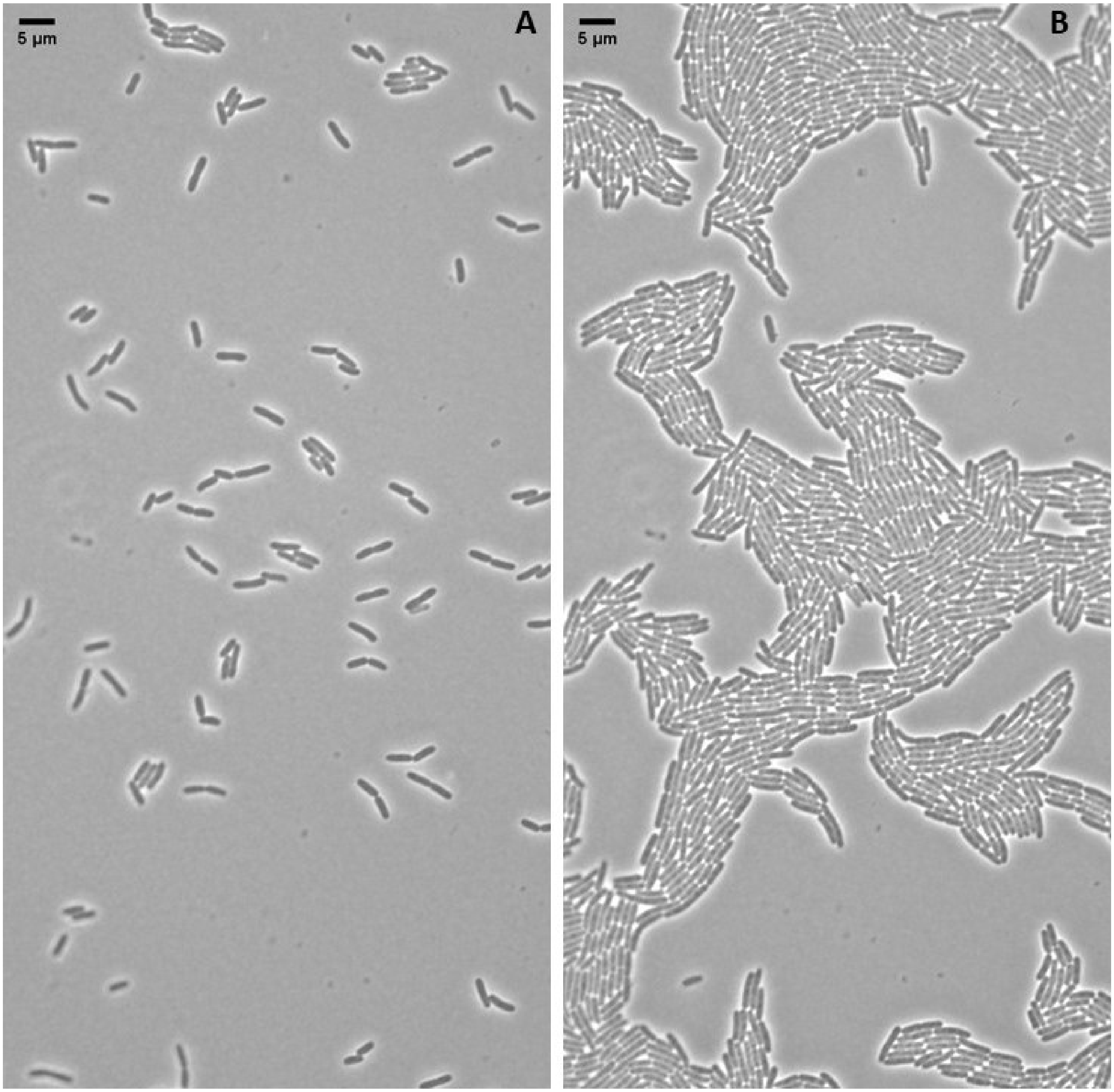

Fig. S6. (A) First and (B) last (100th) images in a time-lapse of unfiltered *E.coli* growth in an unconstrained environment. The initial frame contains 105 cells. The population grows to 1140 by the final frame. Cells touching the edges are not counted. The cells are in monolayer throughout 150 minute duration

F. Unfiltered *E.coli* time-lapses.
